# Case report: Giant lymph node metastases: a new opportunity for cancer radioimmunotherapy?

**DOI:** 10.3389/fimmu.2024.1357601

**Published:** 2024-01-29

**Authors:** Yuan Bian, Zhenhua Zhang, Xiangyu Deng, Qinglian Wen, Dan Li

**Affiliations:** ^1^ Department of Oncology, The Affiliated Hospital of Southwest Medical University, Luzhou, Sichuan, China; ^2^ Department of Radiation Oncology, Cancer Center, West China Hospital, Sichuan University, Chengdu, Sichuan, China

**Keywords:** cervical cancer, lymph node metastasis, radiotherapy, immunotherapy, case report

## Abstract

**Background:**

Despite the significant progress made in radiotherapy and chemotherapy for the treatment of cervical cancer, patients with lymph node metastasis still have a poor prognosis. It is widely accepted that lymph node metastasis plays a crucial role in the spread of cancer to other organs and is considered an independent factor in predicting a poor prognosis. However, recent research suggests that the importance of lymph nodes in tumor therapy needs to be reevaluated, as preserving the integrity of lymph nodes before immunotherapy can enhance treatment effectiveness.

**Case presentation:**

In this report, we present two cases of advanced cervical cancer patients with giant metastatic lymph node lesions in the neck. These patients were effectively treated with a combination of local radiotherapy and immunotherapy after conventional chemoradiotherapy had failed. The combination therapy resulted in significant clinical improvements, with patient 1 achieving over 12 months of progression-free survival (PFS) and patient 2 maintaining sustained remission for an impressive 24 months.

**Conclusions:**

The combination of local radiotherapy and immunotherapy shows promise as a viable treatment option for cervical cancer patients with distant lymph node metastasis, and the giant lymph node metastases may play an important role in this process, which might provide a new opportunity for cancer radioimmunotherapy.

## Introduction

Cervical cancer is the fourth most prevalent malignant tumor and the fourth leading cause of cancer-related deaths in women worldwide (1). Despite advancements in radiotherapy and chemotherapy, metastatic cervical cancer still has a poor prognosis, with a median survival period of 8-13 months and a 5-year survival rate of 16.5% (2). Therefore, there is an urgent need for new therapeutic strategies to improve the outcomes.

Generally, lymph node metastasis (LNM) is considered a critical step in systemic metastasis and plays a crucial role in the spread of cancer. However, some studies have shown that LNM and distant organ metastasis are not always interconnected and the tumor cells may have different origins or metastatic potentials (3, 4). With the emergence of immunotherapy, the significance of lymph nodes in tumor development and antitumor therapy has received considerable attention. In fact, recent research has demonstrated that preserving lymph nodes prior to immunotherapy improves outcomes for solid tumors (5), which might revolutionize conventional treatment strategies.

It is currently believed that any LNM beyond the regional lymph node area would be considered a distant metastasis (M1). In the case of cervical cancer, if there are distant LNMs without other distant organ metastasis, it would fall under stage IVB (6). However, this represents a unique group of advanced patients, and it is worth studying whether they differ from others in terms of treatment options and prognosis. In this study, we present two cases of advanced cervical cancer with giant metastatic lymph node lesions in the neck. The short diameter of distant LMN in both patients exceeds 4cm, accompanied by significant necrosis. These cases were successfully treated with a combination of local radiotherapy and immunotherapy after conventional chemoradiotherapy had failed.

## Case presentation

### Case 1

**Figure 1:**
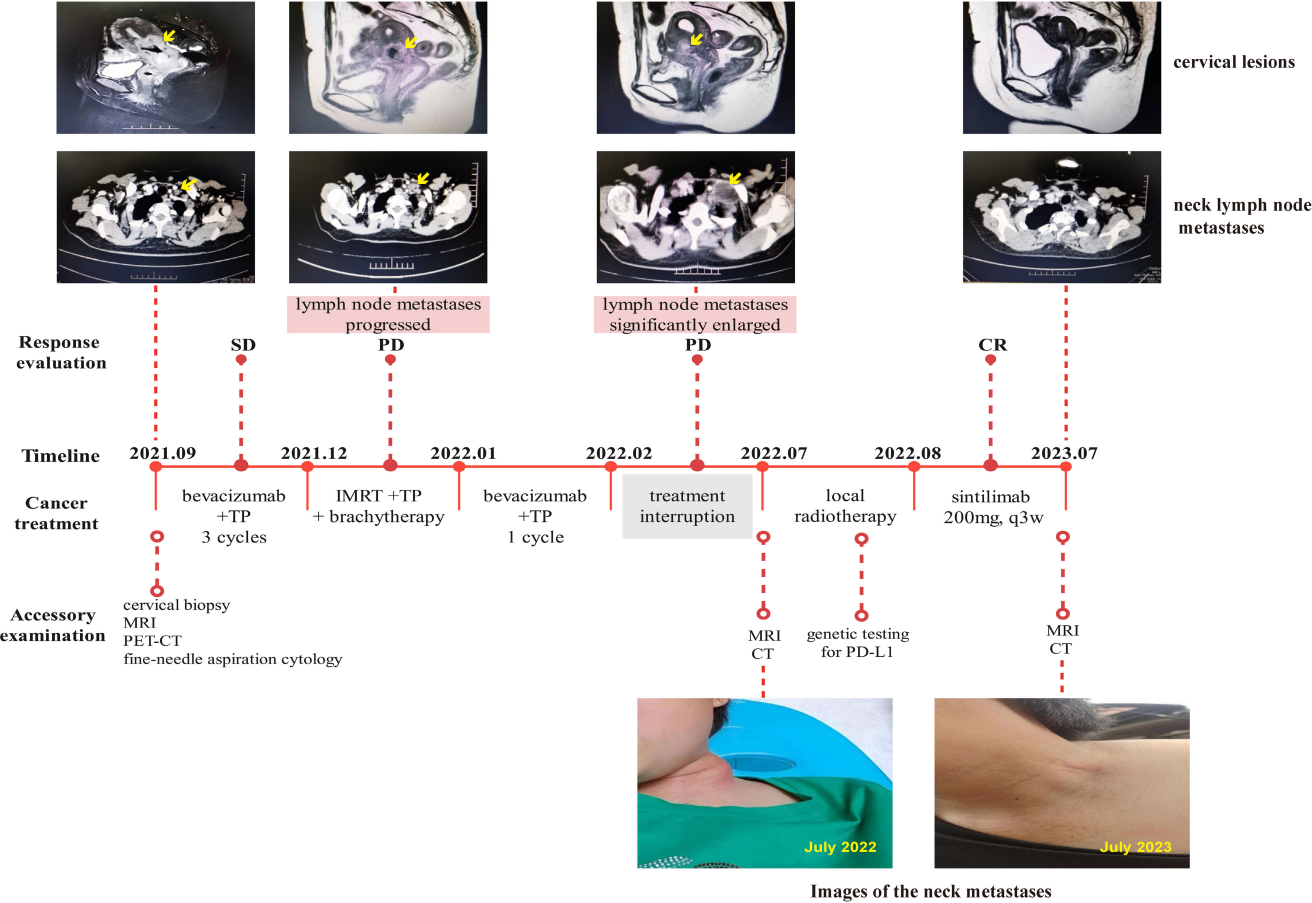
Complete timeline for treatment regimens, disease status, treatment response, and representative images of lymph node metastases for patient 1.

A 45-year-old woman presented with irregular vaginal bleeding and lower abdominal pain in September 2021. Her family history was unremarkable for any neoplastic disease. A gynecological examination revealed that the diameter of the cervix is approximately 3.5cm. The cervix appeared hard and nodular, with slight erosion of the surface mucosa. The left main sacral ligament was shortened, but there was no significant thickening. The uterus was enlarged, resembling changes seen on the 50th day of pregnancy. It had clear boundaries, was mobile, and exhibited mild tenderness. There were no palpable obvious enlarged lymph nodes in the bilateral neck and clavicle. After colposcopy and underwent cervical biopsy, the patient was diagnosed with poorly differentiated adenocarcinoma of the cervix. The PET-CT scan revealed multiple lymph node metastases throughout the body, including the left supraclavicular lymph node. This was later confirmed by fine-needle aspiration cytology, resulting in the identification of stage IVB. The patient was treated with bevacizumab in combination with the TP chemotherapy regimen (paclitaxel + cisplatin). After three cycles of treatment, the curative effects were evaluated as stable disease (SD). Subsequently, the patient underwent pelvic external beam radiotherapy (EBRT) (50Gy in 1.8Gy/fraction) with one cycle of concurrent TP chemotherapy, followed by brachytherapy (28Gy in 7Gy/fraction). However, one month after the therapy, the neck lymph node metastases had progressed, even though the cervix lesions had shrunk. Despite our recommendation for local radiotherapy for the metastases, the patient chose to discontinue treatment for personal reasons after receiving another cycle of bevacizumab along with the TP regimen. In July 2022, five months after the last treatment, the patient sought medical attention again due to the noticeable growth of lymph node lesions, pain, and significant weight loss. On the left clavicle, a hard, fixed, and slightly tender lymph node with a size of about 4cm can be palpated, with redness and swelling. Gynecological examination showed an atrophic change in the cervix, with a slight thickening and shortening of the left parametrial sacral ligament on vagino-recto-abdominal examination. CT and MRI scans revealed the progress of supraclavicular lymph node metastases, with persistent cervical lesions (**Figure 1**). To alleviate symptoms of local lymph node compression, the patient received local radiotherapy (70Gy in 2Gy/fraction) and underwent genetic testing for programmed death-ligand 1 (PD-L1), which revealed a combined positive score (CPS) of 6. Due to economic reasons, the patient was unable to choose pembrolizumab for immunotherapy. As a result, sintilimab (200mg, q3w), a recombinant humanized anti-PD-1 monoclonal antibody (anti-PD-1mAb) developed in China, was continued as maintenance therapy after radiotherapy. The use of sintilimab as a treatment for cervical cancer had not been approved in China. However, we obtained informed consent from the patient and filed it with the hospital, as we were administering the drug beyond its approved indications. The patient’s last follow-up in July 2023 showed no disease progression (shown in **Figure 1**), and the PFS was more than 12 months, which is particularly encouraging.

### Case 2

**Figure 2:**
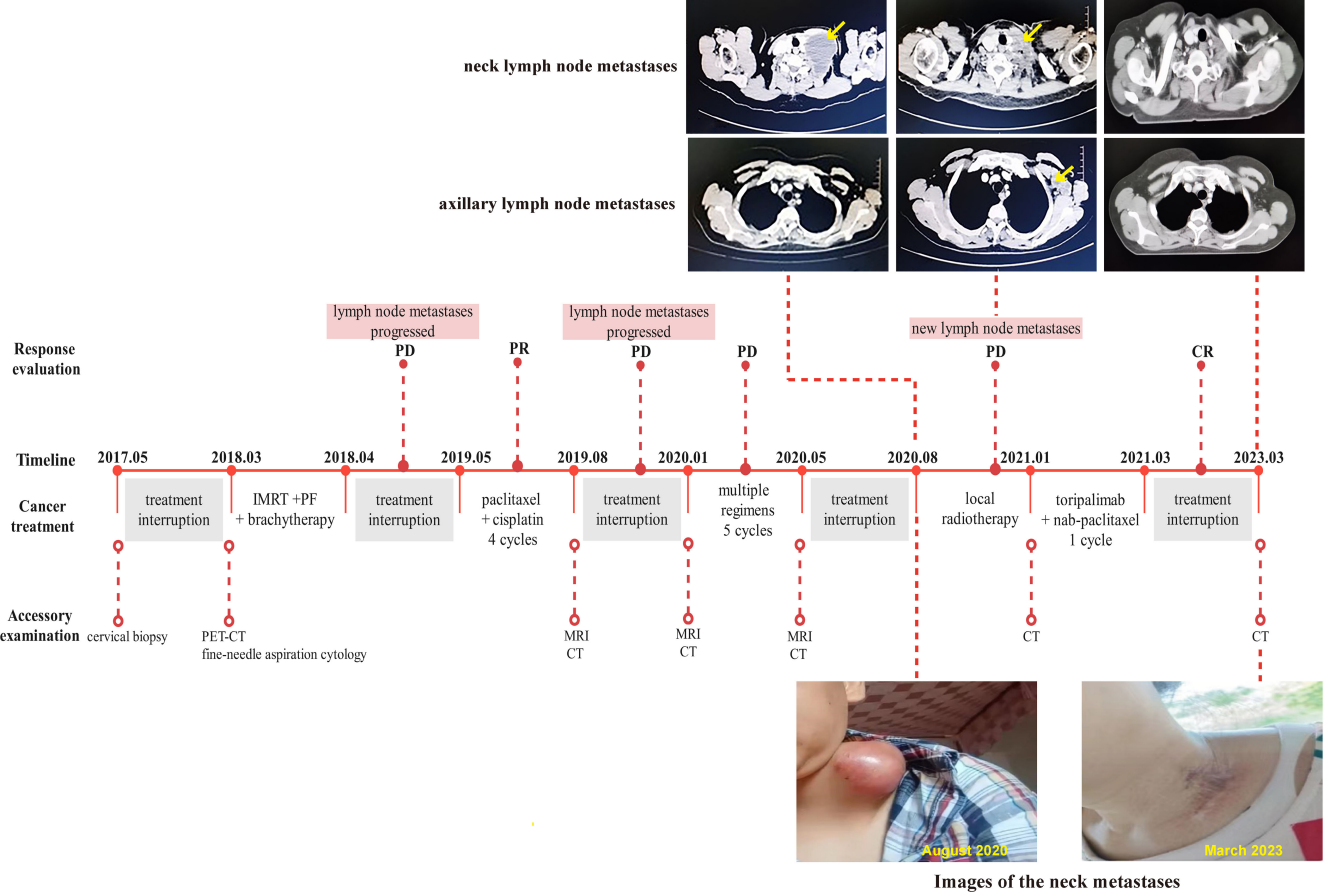
Complete timeline for treatment regimens, disease status, treatment response, and representative images of lymph node metastases for patient 2.

A 48-year-old woman was admitted to our hospital in May 2017 due to irregular vaginal bleeding, with negative cancer family history. A biopsy confirmed squamous cell carcinoma of the cervix, but the patient declined further treatment. In March 2018, the patient returned due to severe anemia and PET-CT imaging revealed metastases in multiple lymph nodes, including the left supraclavicular lymph node. A gynecological examination revealed the presence of a new organism in the cervix, resembling a cauliflower and measuring approximately 4cm in diameter. This organism is affecting the left vaginal fornix and is accompanied by left parametrial involvement. Additionally, an enlarged lymph node measuring approximately 1cm in diameter can be palpated on the left clavicle. After the management of bleeding with arterial embolization, the patient underwent EBRT (45Gy in 1.8Gy/fraction), as well as two cycles of concurrent chemotherapy with PF regimen (fluorouracil + cisplatin), and then brachytherapy (30Gy in 6Gy/fraction). However, the patient refused local radiotherapy for the metastases, leading to the discontinuation of treatment. In May 2019, one year after the last treatment, the patient was readmitted to the hospital due to growing swelling of the supraclavicular lymph nodes. A hard mass approximately 8cm in size was found above the collarbone, with redness and swelling on the surface. The lesion significantly shrank after 4 cycles of treatment with the TP regimen, but the patient once again declined local radiotherapy. About 5 months later, the patient returned to the clinic with the same symptoms, and multiple changes were made to the chemotherapy regimens due to poor efficacy, including the combination of anti-angiogenic drugs. To alleviate symptoms, regional lymph node radiation (70Gy in 2Gy/fraction) was performed in August 2020. Unfortunately, three months later, a left axillary lymph node metastasis was found, despite a considerable decrease in the metastases to the supraclavicular lymph node according to the enhanced CT scan (shown in **Figure 2**). Therapeutic evaluation as progressive disease (PD). Considering the poor efficacy of previous chemotherapy and anti-vascular targeted therapy, after communicating with the patient, chemotherapy combined with immunotherapy was chosen. The patient refused genetic testing. Due to economic reasons, the patient was unable to choose pembrolizumab, which has indications for the treatment of recurrent or metastatic cervical cancer. Following the administration of one cycle of nab-paclitaxel (260mg/m^2^) combined with toripalimab (240mg), a novel humanized IgG4 monoclonal antibody against PD-1 developed in China, the patient discontinued further treatment and regular follow-up due to financial reasons in March 2021. The use of toripalimab as a treatment for cervical cancer has not been approved in China, and we have signed an informed consent form with the patient and filed it with the hospital for exceeding the instructions. The patient was not followed up regularly afterward. Fortunately, the patient returned to our hospital in good health after two years with no other treatment being given. Lesions in the neck and axillary area disappeared on the CT scan, and the patient showed a complete response (CR) to the treatment at the last follow-up in March 2023. As of that time, the PFS had increased to an encouraging 24 months.

## Discussion

The prognosis for individuals with advanced metastatic cervical cancer is often very poor due to a lack of effective treatments. Typically, chemotherapy and radiotherapy are used, but the benefits are not always long-lasting (7, 8). In recent years, bevacizumab, an anti-angiogenic agent used in oncology therapy, has emerged as a new treatment option for advanced cervical cancer. Combining bevacizumab with the standard chemotherapy regimen has significantly improved PFS and overall survival (OS) (9–11). However, many patients still experience disease progression shortly after treatment, with a PFS of 8.2 months and a response rate (RR) of 48% (9). Therefore, there is an urgent need for new therapeutic strategies.

Immunotherapy is a promising therapeutic approach in tumor therapy, with immune checkpoint inhibitors (ICIs) showing encouraging antitumor activity in various solid tumors (12, 13). This provides new hope for the treatment of advanced cervical cancer, which is frequently associated with high-risk HPV virus infection and the up-regulation of PD-L1 expression (14, 15). Evidence of an endogenous anti-tumor immune response in cervical cancer is increasing, indicating the potential of immunotherapy in this context (16–18). Pembrolizumab, authorized by the FDA in 2018, has shown effectiveness in treating recurrent or metastatic advanced cervical cancer expressing PD-L1 (CPS≥1) based on the KEYNOTE-158 clinical study (19). It has also been authorized for patients with high tumor mutational load (TMB≥10 mutations/Mb) or high microsatellite instability (MSI-high). Sapalizumab was licensed by the National Medical Products Administration (NMPA) of China in September 2023 as the first anti-PD-1 monoclonal antibody in China for treatment in patients with recurrent or metastatic PD-L1-expressing cervical cancer who had previously failed platinum-containing chemotherapy (20). Additionally, ipilimumab, an antibody against cytotoxic T-lymphocyte-associated protein 4 (anti-CTLA-4 mAb), has also demonstrated good tolerance and safety (21, 22). In June 2022, the NMPA approved cadonilimab, a bispecific antibody targeting PD-1 and CTLA-4, as a potential new-generation anticancer drug for persistent, recurrent, or metastatic cervical cancer, with an ORR of 42.9% and a CR of 17% in advanced patients with PD-L1-positive expression (23). Besides, it has been demonstrated that the level of TGF-β increases as normal cervical tissues progress to malignant tumors, and the tumor microenvironment (TME) contains a significant amount of TGF-β in cervical cancer. Consequently, anti-PD-(L)1/TGF-β bispecific antibodies like M7824, YM101, and TST005 have a broad range of applications in enhancing the therapeutic effectiveness for patients with cervical cancer (24–26). However, it is important to note that the response rates to single ICI treatment for cervical cancer are relatively low, ranging from 12.5% to 26% (19, 27, 28).

When a tumor develops LNM, it is often considered a significant factor contributing to poor prognosis. However, lymph node dissection does not seem to significantly improve patient prognosis in certain tumors (29–31). Moreover, there is still no consensus on whether LNM affects the metastasis of distant organs. It has been hypothesized that lymph nodes exhibit an anti-tumor metastasis phenotype during homeostasis. However, when stimulated by certain factors, the tumor microenvironment (TME) undergoes changes that inhibit the anti-tumor immune response and promote tumor metastasis (32, 33). Studies have reported immunological dysregulation and decreased immune response, such as dysfunction in antigen presentation, depletion of CD8+ T cells, and accumulation of Treg cells, in tumor-draining lymph nodes (TDLN) (33, 34). On the other hand, TDLN presents a significant opportunity for therapeutic intervention to prevent local and distant metastasis through reactivation of the antitumor immune response. This highlights the importance of maintaining lymph node integrity before immunotherapy (5, 35). Recent trials have examined the therapeutic potential of using immunotherapy as a neoadjuvant treatment prior to surgery, showing promising results (5). Simultaneously, combining immunotherapy with radiotherapy seems to be a proven strategy to generate anti-tumor reactivation in TDLN.

Radiotherapy is traditionally considered a local treatment, but recent research has brought attention to its immunomodulatory effect. Studies have shown that radiotherapy can enhance the formation of tumor-associated antigen-MHC complexes, promote antigen cross-presentation, increase T-cell infiltration, and stimulate the production of antitumor antibodies by B-cells (36, 37). However, radiotherapy also leads to an upregulation of PD-L1 expression on tumor cells, which inhibits the immune response (38). To address this issue and to improve treatment outcomes, radiotherapy combined with PD-L1 inhibitors is a promising strategy. In the NiCOL phase 1 clinical trial, the combination of nabulizumab with concurrent chemoradiotherapy (CRT) followed by maintenance therapy demonstrated a favorable safety profile and promising results for locally advanced cervical cancer (39). Additionally, research has shown that sequential ipilimumab therapy following CRT was well-tolerated and effective in patients with lymph node-positive cervical cancer, and combining immunotherapy with CRT was more effective than conventional CRT (22). Although the combination strategies of radiotherapy and immunotherapy have made significant progress, there are still several unresolved issues before their widespread implementation in clinical practice. These include the determination of optimal radiotherapy dosage and fractionation, the selection of combination regimens and administration order, the identification of the most suitable duration for immunotherapy, and so on (40).

To the best of our knowledge, this is the first report of local radiotherapy combined with immunotherapy for cervical cancer with giant neck lymph node metastasis, yielding promising results. In our study, patients who had previously undergone conventional chemoradiotherapy, even along with targeted anti-angiogenic treatment, experienced disease progression. Fortunately, when local radiotherapy was combined with immunotherapy, satisfactory outcomes were achieved. When the patients receive immunotherapy, pabolizumab is the only PD-1 inhibitor approved by the FDA for cervical cancer. However, its high price makes it unaffordable for many patients in China. After obtaining the patient’s fully informed consent as well as approval from our hospital, we chose the comparable drugs that were affordable to the patients. Real-world data confirms that this same mechanism of drug substitution still has certain therapeutic effects, and the safety is controllable. The patients remain in good health and achieve complete remission for a significant duration. Patient 1 received immunotherapy immediately after radiotherapy and had a PFS of more than 12 months at the last follow-up. Patient 2 experienced localized regression of the neck lesion three months after radiotherapy but developed a new lesion in the axillary lymph node. After receiving one cycle of sequential immunotherapy combined with chemotherapy, she discontinued treatment. Two years later, a CT scan revealed the complete disappearance of the lesions, not only cervical lymph node metastases but also axillary lymph node metastases that were not in the irradiation field. Patient 2 achieved a PFS of more than 24 months, despite receiving only one course of immunotherapy.

## Conclusions

In conclusion, our patients were very satisfied with the good therapeutic effect, the very limited side effects, and the affordable financial burden. Both patients showed significant weight gain. When lymph nodes in the neck metastasize to a certain volume, it becomes one of the main symptoms that trouble patients. Administering radiation therapy alone may not completely eradicate the local lymph nodes due to the possibility of enlarged lymph nodes coexisting with tumor necrosis. However, when combined with immunotherapy, it can transform the original local palliative treatment into curative treatment. This combined approach not only eliminates local lymph node metastases but also targets distant lesions that are not within the radiation therapy area. The immune antigen role played by the massive lymph node metastasis may be crucial for the sustained therapeutic effect of this combined mechanism. Future clinical trials should investigate the significance of preserving lymph node integrity for immunotherapeutic treatment and explore the role of giant lymph node metastases in combination radiotherapy and immunotherapy.

## Data availability statement

The original contributions presented in the study are included in the article/Supplementary Material. Further inquiries can be directed to the corresponding authors.

## Ethics statement

Written informed consent was obtained from the individual(s) for the publication of any potentially identifiable images or data included in this article.

## Author contributions

YB: Data curation, Formal analysis, Writing – original draft. ZZ: Data curation, Formal analysis, Writing – original draft. XD: Formal analysis, Writing – original draft. QW: Conceptualization, Writing – review & editing. DL: Conceptualization, Writing – review & editing.
